# Therapeutic Horticulture as a Therapeutic Intervention in Patients Receiving Electroconvulsive Therapy (ECT) Treatment for Major Depressive Disorder

**DOI:** 10.7759/cureus.17363

**Published:** 2021-08-22

**Authors:** Tessy Korah, Deborah Morrison, Dana Mason, Elizabeth R Diehl, Regina Bussing

**Affiliations:** 1 Psychiatry, University of Florida, Gainesville, USA; 2 Environmental Horticulture, University of Florida, Gainesville, USA

**Keywords:** major depressive disorder, therapeutic horticulture, electroconvulsive therapy, quality of life, adjunct therapy

## Abstract

This study explored the effect of a structured therapeutic horticulture (TH) program on depression symptoms and quality of life indicators for individuals receiving inpatient electroconvulsive therapy (ECT) for major depressive disorders (MDD). Self-reported measures of depressive symptomatology (PHQ9, BDI-II) and quality of life (SF-36) were employed to compare intervention (*n* = 25) and control groups (*n* = 27), with the intervention group attending TH sessions for one-hour periods, twice per week, in addition to standard inpatient care associated with ECT received by both groups. All patients were assessed at admission, and after two weeks’ time or prior to discharge, during which the intervention group participated in a minimum of four TH sessions. Sessions were led by a horticultural therapist in an accessible on-campus greenhouse. Both groups improved significantly between assessment times one and two on both measures of depression, with a statistically significant difference in change scores for the BDI-II only, favoring the control over the intervention group (16.5, *s.d. *12.78 versus 9.6, *s.d. *10.15; *p* = 0.36). Both groups improved significantly on four of eight SF-36 subscales during the same period. A statistically significant difference in change scores was found for the Role Limitations-Physical Health (RLPH) subscale, where the intervention group improved between assessment periods, whereas the control group worsened (16.0, *s.d.*48.8 versus -9.3, *s.d.* 33.4; *p* = .033). Although quantifying group changes or improvement for individuals receiving intensive treatment for major depressive disorders (ECT) by the addition of an adjunct therapy is difficult, this study provides a basic premise for the consideration of various therapeutic horticulture settings to achieve therapeutic benefits through TH.

## Introduction

According to the National Institute of Mental Health, depression is one of the most common mental disorders suffered by Americans every year. The 2017 National Survey on Drug Use and Health revealed that an alarming 17.3 million adults in the US experienced a major depressive episode, with 8.7% of women and 5.3% of men suffering from the disorder [1].

The Diagnostic and Statistical Manual of Mental Disorder 5th Edition (DSM-5) describes major depressive disorder (MDD) as a mental illness comprising a period of two weeks or more in which there is a lack of interest in activities and/or a depressed mood [2]. There must also be present four other symptoms that affect sleep, energy, body image, self-esteem, and concentration. Depression may result in outpatient treatment for less severe cases and inpatient treatment for more debilitating instances. Inpatient treatment for severe MDD is common and in many cases, admission occurs due to suicidal ideation or after a suicide attempt.

Medical professionals have sought to develop treatments to properly address and alleviate the symptoms of depression. Treatment guidelines issued by the American Psychiatric Association refer to common and empirically proven treatments, including antidepressant medication and psychotherapy, as well as neuromodulation treatments such as transcranial magnetic stimulation (TMS) and electroconvulsive treatment (ECT) [3]. Professionals have also looked for alternative therapies and supplemental activities to aid those suffering from depression. Among them is therapeutic horticulture.

Therapeutic horticulture (TH) is a process that uses plant-related activities to improve patient wellbeing through full, partial, or passive participation [4]. This process uses horticulture activities and nature interaction as a therapeutic modality to support program goals and has been proposed as early as 1979 as a therapeutic adjunct strategy for various physical and mental ailments [5]. Although primarily suggested to have a role in overall stress reduction in adults and older adults, TH also has been shown to promote stress reduction in children as measured by a number of physiological criteria that include skin conductance, sympathetic nervous activity, and parasympathetic nervous activity as indicators of wellness [6-8]. Moreover, in palliative care settings, TH appeared to have promoted an overall positive experience for participating patients as well as for palliative care teams [9-10].

Research has shown that time spent in nature can result in increased positive mental health. Nature, as defined here, includes seeing the outdoors from a window, exercising in nature, or simply sitting outdoors in a green space. Self-esteem and mood have been evaluated in green-exercise research and have shown that regularly spending time in nature is related to increased lifespan and a lower risk of mental illness [11]. A review of seven intervention studies with pretest/post-test design and a wide range of cohort sizes, ranging from eight to 129, which explored the impact of TH in older adults with dementia has shown clear improvements in patient well-being [12]. A two-year TH study involving dialysis patients resulted in significant improvement in physical functioning following intensive involvement in a TH program [13]. Further, a 12-week-study combining TH with antipsychotic medications has shown a statistically significant alleviation of psychiatric symptoms in patients with schizophrenia [14]. However, studies that systematically explore the effectiveness of TH as a standalone or supplementary approach for people with mental illness are scarce [15-16].

Few studies to date have assessed the role of therapeutic horticulture on depression, but the research that has been collected thus far in this area points to benefits for patients with depression [17] and anxiety [17-18]. Gonzalez and colleagues [19] found that a sample of 28 patients presenting with clinical depression who attended a 12-week program had clinically significant reductions in their Beck Depression Inventory Scale scores. Attentional Function Index (AFI) scores that measure perceived effectiveness in common activities requiring attention and working memory, specifically the ability to formulate plans, carry out tasks, and function effectively in daily life under normal circumstances also increased as a result of participating in the horticulture program [20]. Although TH has been used for decades [19], research examining its’ effects on mental health is limited. No literature could be identified on the potential therapeutic effects for patients with MDD.

This current pilot study seeks to examine the impact of TH on patients with moderate and severe MDD, delivered in addition to empirically supported treatments, namely, ECT with or without psychotropic medications in an inpatient setting. The study delivers a non-randomized supplemental intervention (therapeutic horticulture) while all participating patients also receive their usual care as determined and delivered by their treating inpatient physician. The design is non-randomized because it was logistically too challenging to conduct this pilot study with only one-half of the inpatients. Instead, comparison data were obtained for patients receiving MDD treatment as usual, when the horticultural intervention was not offered on the unit. Specifically, we examine changes in two scales measuring depression symptoms (Beck Depression Inventory-II [21], Patient Health Questionnaire-9 [22]) and in a Quality of Life measure (Health Survey 36-Item Short Form) [23] in patients with MDD over the course of up to two weeks of treatment with and without therapeutic horticulture. We hypothesized that depression ratings would decrease and quality of life would increase during these two weeks in both groups and that the scope of improvement in both measures would be larger when TH was added to treatment.

## Materials and methods

Institutional review board approval was obtained prior to the recruitment of the study cohort. The study was conducted in the inpatient units of a university hospital psychiatry department and in the in-campus horticulture facility affiliated with the same large public university. All participants provided informed consent prior to beginning study participation. Within consent language for the control group, patients seeking treatment, including electroconvulsive therapy (ECT), psychotherapy, and use of prescribed psychotropic medications for moderate or severe depressive disorders, were informed that the purpose of the study was to evaluate the reduction in depressive symptoms and perception of overall quality of life during psychiatric inpatient treatment. Measures would be completed within three days of admission and again after two weeks or upon discharge (whichever came earlier).

Within the consent language for the intervention group, patients were informed that the purpose of the study was to evaluate the effectiveness of TH as an adjunct to ECT therapy, and/or psychotherapy, and/or use of psychotropic medications in alleviating depressive symptoms and increasing perception of quality of life. Patients were informed that they would attend two sessions per week of one hour each at the gardens and greenhouse, and that during these sessions, they would learn to grow, maintain, and propagate plants, including vegetables, herbs, tropicals, and succulents. For this group, the same set of measures would be completed at the start of the TH program, again after attending two weeks (four sessions) of TH programming, and again upon exiting the study. Patients admitted for ECT treatment were invited to attend the TH programming on a rolling basis according to the next scheduled TH session after agreeing to participate in the study.

Inclusion criteria

Patients aged 18 and over were required to have been diagnosed with moderate or severe major depressive disorder (MDD), have undergone multiple episodes, to be voluntarily in treatment for their condition, and to be able to consent for themselves to participate. Patients with active psychosis, including schizophrenic spectrum disorders, active substance use, or moderate to severe dementia, were ineligible to participate. No additional factors, including physical limitations, were considered for exclusion.

Recruitment

Members of the research team working on the inpatient unit reviewed the relevant information of newly admitted patients to identify those who met study criteria, approached eligible patients, introduced the study, and inquired about interest in participating. Informed consent was gained from patients agreeing to participate. Student volunteers who were unaware of their future designation into intervention and control groups assigned the recruited cohort alternatively to two groups. The final analyzed cohort consisted of 25 patients in the TH intervention and 27 patients participating in the control condition without TH. Both intervention and control groups received standardized pharmacological as well as ECT treatments during the course of the study.

Intervention

Therapeutic horticulture sessions were held twice each week for one hour per session, over a 14-week period. Patients who were expected to remain on the inpatient unit for more than the number of days required to complete four TH sessions (minimum of 11 days) and the requisite measures were invited to participate for the duration of their stay. Greenhouse sessions were led by an American Horticultural Therapy Association (AHTA)-registered horticultural therapist (LD) who was assisted by specially trained volunteers, many of whom were also master gardeners.

Study participants took part in plant propagation, cultivation, and maintenance, as well as in extensions of those activities such as sensory interactions, horticulture education, and plant crafts. Many activities are built on each other to provide session continuity and to help participants experience the various stages of horticulture and the uses of diverse plant materials. For example, in one session, participants could choose herb or vegetable seeds to plant. In a subsequent session, once the seedlings had emerged, participants learned about the importance and techniques of thinning seedlings and practiced with their set of seedlings. In later sessions, participants transplanted their young plants into pots. Participants were encouraged to take their plants and projects home when discharged and were provided with information for their ongoing care.

Measures 

Beck Depression Inventory (BDI-II)

The BDI second edition [21] is made up of a 21-item self-administered survey that takes anywhere from five to 10 minutes to complete. It is scored by adding ratings given for each item and measures depression severity. Scores range from 0 to 63 with categorical results based on item totals and resulting in the following depression levels: minimal (≤ 13); mild (14-19); moderate (20-28), and severe (29-63). Excellent levels of internal reliability (21 items; α = .92), as well as for test-retest correlation (r = .93) are reported [21].

Patient Health Questionnaire (PHQ-9)

The PHQ-9 [22] is a nine-item self-administered survey that generally takes less than five minutes to complete. It is scored by adding ratings given for each item and measures depression severity. Scores range from 0 to 21 with categorical results and clinical cutoffs (≥10) based on item totals and resulting in the following depression levels: Minimal (≤ 4); Mild (5-9); Moderate (10-14); Moderately severe (15-19), and Severe (21-28). Excellent levels of internal reliability (9 items; α = .89) and test-retest reliability (stat) are reported [23].

Medical Outcomes Study 36-Item Short-Form Health Survey (SF-36)

The SF-36 is a quality of life measure created for use in clinical and research settings [24]. It is a self-administered survey composed of 36 items that evaluate eight health areas, including limitations in physical activities due to physical health problems (Physical Functioning; PF), limitations in usual role activities due to physical health (Role Limits-Physical Health; RLPH), limitations in usual role activities due to emotional problems (Role Limits-Emotional Health; RLEH), levels of energy and fatigue (EF), emotional well-being (EWB), limitations in social activities because of physical or emotional problems (Social Functioning; SF), frequency and interference with usual roles due to bodily pain (Bodily Pain; BP), and perception of general health (General Health; GH). The score for each subscale is the weighted sum calculated from unique questions that comprise each subscale. Scores from each subscale fall between 0 and 100, with higher scores indicating higher functioning within each domain, including fewer limitations to daily life, physical activities, and less pain or emotional problems.

Medical Record-Derived Treatment Data

Patients provided consent to conduct medical record review for current and past psychiatric diagnoses, treatments (including ECT, TMS, psychotropic medications, psychotherapy).

Statistical analysis

All analyses were conducted using SPSS version 27 software (IBM Corp., Armonk, NY). Pearson correlational testing was used to assess the relationship between the two measures of depression (PHQ9 and BDI-II) at each study time point. For each study measure, two sets of analyses were performed: a within-group analysis and a between-groups analysis. Homogeneity of variances was tested and confirmed using Levene’s Test for Equality of Variances. Paired t-tests were used to compare groups on the change between time points in each group. Independent samples t-tests were used to investigate changes between groups. Effect sizes for all tests were computed. Hypotheses tests were two-sided with the level of significance set at .05.

## Results

Twenty-five (25) individuals met the criteria for inclusion of their data in the analysis for the intervention group by completing Time 1 assessments followed by attendance at four TH sessions and completion of assessment measures at Time 2 (Table 1). The intervention cohort consisted of 14 males, 10 females, and one unreported gender, with ages ranging from 32 to 72 years old. The average length of inpatient stay for this group was 13 days, with a range of 11-53 days.

**Table 1:** Characteristics of intervention and control group participants

	Intervention	Control
Participant number	25	27
Age range (in years)	32 - 72	19 - 84
Gender Male Female Unavailable	14 10 1	10 17
Length of stay: mean; range (days)	13; 11-53	7.5; 2-18

Twenty-seven (27) individuals met the criteria for inclusion of their data in the analysis for the control group by completion of the assessment measures at Time 1 and Time 2. The control group patients were 10 males and 17 females ranging in ages from 19 to 84 years old. The average length of inpatient stay for this group was 7.5 days with a range from 2-18 days.

Time 1 ratings, representing patients’ symptoms and quality of life experience upon admission for inpatient treatment, indicated that the majority of study participants scored in the moderate to severe range on the BDI and PHQ9, respectively. Similarly, their quality of life indices indicated significantly lowered quality of life on all subscales, particularly in areas of Role Limits - Emotional Health as well as Physical Health, Energy/Fatigue, Emotional Wellbeing, and Social Functioning. As shown in Table 2, intervention and control group participants improved significantly between assessment Times 1 and 2 on measures of depression (PHQ9 and BDI-II), with large effect sizes (d = 1.27 and .94 for intervention, and d = 1.20 and 1.29 for control participants, respectively). There was a statistically significant difference favoring control over the intervention group on change in depression at Time 2 as measured using the BDI-II (p = .036); however, the control group’s Time 1 BDI-II score started out higher than the intervention groups (36.7 versus 30.4, respectively). The intervention and control groups improved significantly on four of eight subscales of the SF-36 measure during the same period. Both groups showed improvements in Energy/Fatigue, Emotional Wellbeing and Social Function of medium effect size (d = .65, .66 and .50 for intervention group and d = .50, .48 and .53 for control, respectively). The intervention group also showed significant improvements in General Health ratings (d = .51) and the control group for Role Limits - Emotional Health (d = .55). The intervention and control groups differed in their change in the Role Limitations-Physical Health (RLPH) subscale between assessment periods (p = .033), with the intervention group showing improvements, whereas the control group showed deterioration.

**Table 2:** Comparison of assessment measures Legend: Time 1 = admission; Time 2 = end of second week 2; ES = effect size (within group change); *, ** = Within group means significantly improved from Time 1 to Time 2 on these measures *(p = <.001), **(p = < .05); ns = not significant PHQ-9: Patient Health Questionnaire; BDI-II: Beck Depression Inventory; SF-36: Medical Outcomes Study 36-Item Short-Form Health Survey

	Intervention Group (N=25)			Control Group (N=27)			Between-Group Change
	Time 1	Time 2	ES	Time 1	Time 2	ES	
Depression Measures	Mean (SD)	Mean (SD)		Mean (SD)	Mean (SD)		
PHQ-9	20.00 (5.01)	11.84 (6.45)*	d = 1.27	20.63 (5.60)	10.85 (6.71)*	d = 1.20	ns
BDI-II	30.44 (12.24)	20.88 (13.36)*,*	d = .94	36.70 (10.22)	20.22 (12.83)*	d = 1.29	p = .036
SF-36 Subscales							
Physical Functioning (PF)	54.40 (25.75)	61.60 (29.52)	d = .27	57.41 (31.60)	64.44 (31.82)	d = .31	ns
Role Limits-Physical Health (RLPH)	22.00 (35.59)	38.00 (43.37)	d = .33	34.26 (43.38)	25.00 (34.67)	d = .28	p = .033
Role Limits-Emotional Health (RLEH)	6.67 (21.52)	22.67 (39.35)	d = .38	0.00 (0.00)	19.75 (36.11)**	d = .55	ns
Energy/Fatigue (EF)	19.40 (17.99)	35.20 (26.67)**	d = .65	23.15 (19.57)	35.37 (21.26)**	d =. 50	ns
Emotional Well Being (EWB)	26.40 (18.26)	41.60 (23.97)**	d = .66	29.78 (15.82)	40.15 (19.49)**	d = .48	ns
Social Functioning (SF)	18.50 (21.38)	33.00 (28.16)**	d = .50	18.98 (18.47)	36.57 (27.50)**	d = .53	ns
Bodily Pain (BP)	49.30 (30.59)	54.50 (27.16)	d = .18	61.02 (32.83)	58.24 (33.63)	d = .12	ns
General Health (GH)	41.00 (20.56)	52.60 (23.14)**	d = .51	43.89 (24.94)	50.93 (23.21)	d = .30	ns

## Discussion

This pilot study sought to explore whether patients suffering from MDD and treated with ECT may experience further improvements in depressive symptoms and quality of life through the addition of therapeutic horticulture. Results show that patients experienced significant improvement in their mood symptoms as assessed by PHQ-9 and BDI-II during their course of ECT treatment and that the addition of TH yielded no sizeable additional improvement on these outcome measures. A slightly larger improvement of BDI-II scores in the control group was observed but may be a function of higher BDI scores on admission in this group, with a larger opportunity for improvement, as both groups had comparable BDI-II scores at Time 2. These findings are consistent with other studies reporting significant improvements through inpatient ECT treatment for patients suffering from MDD and suggest that detecting additional improvements associated with receipt of less intensive treatment, such as TH, cannot be achieved through standard measures of depression.

Nevertheless, further study of depression treatment enhancement, including with modalities such as TH, is called for because MDD has been recognized as the leading cause of disability and affected patients clearly show impairments in the quality of daily life; physical, psychological, social functioning; and overall well-being [25-26]. Therefore, it remains crucial to determine which treatments are most efficacious for what patient populations and whether antidepressant regimens should be sequenced or combined to achieve recovery in the shortest time period. Although antidepressants are traditionally used first to treat MDD, some recent studies suggest that ECT could be considered a first-line therapy for MDD due to its profound effects [27]. Some studies showed improvement in patient’s symptoms in response to ECT independent of the use of antidepressants [28-29]. When combined with antidepressants, ECT also showed enhancement in the medication’s antidepressant effects [30], and did not influence the cognitive outcomes of antidepressants [31-32]. A recent study that specifically focused on the effects of ECT on health-related quality of life (HRQOL) showed that patients receiving ECT with antidepressants had better HRQOL on every dimension of the SF-36 compared with patients who received antidepressants alone [33].

Our study adds to the literature on the role of TH in depression treatment by expanding to a severely affected inpatient population with MDD. Although earlier small group studies addressing a potential role for TH in depression treatment showed a positive trend towards improvement in overall clinical symptoms [34-35], a clear and significant effect of TH on clinical depression was not evident until structured studies addressing specific groups, such as geriatric patients and those with cognitive impairments, were conducted [36-39].

It has been widely recognized that the mood symptoms of MDD are also associated with substantial impairments in health-related quality of life, which ultimately will interfere with the patients’ ability to take care of everyday responsibilities [40-41]. In this regard, antidepressants, as well as ECT, are of significant value [41-43] except in cases of treatment-resistant MDD (TR-MDD) [43]. Adding anesthetics such as ketamine is found to improve the HRQOL in such cases of TR-MDD [43-44]. Results of the current study suggest the merits of further study of alternate approaches, including TH, along with the standard ECT for treating TR-MDD, considering the improvement in the Role Limitations-Physical Health (RLPH) subscale in the intervention group patients who received TH compared to the control group who received ECT/antidepressants.

This study has several limitations. First, the relatively small cohort size limits the power to explore specific effects on various MDD severity subgroups as well as gender and age differences. Also, we did not account for the potential impacts of antidepressant medication regimens the patients may have been on concurrent with ECT. Further, the ability to assess the impact of standard TH intervention was most hampered by the length of stay (LOS) for patients in both study groups. Given the usual short inpatient LOS, we a priori determined that four greenhouse sessions over two weeks would represent therapeutic TH dosing. More extensive TH dosing might have produced more sizeable effects. Our IRB protocol allowed for patients who stayed longer than two weeks to continue in TH sessions, but only four patients remained on the inpatient unit long enough to attend additional TH sessions with subsequent assessment. These numbers were simply insufficient to allow for quality data analysis of outcomes associated with more extended TH. Many patients in the TH cohort reported enjoyment from the TH experience, and a small number of discharged patients even took up the IRB-approved option to continue as outpatients in the TH program. However, as other environmental variables could not be controlled, data was not collected from this small group, as they would not be valuable for analysis. Additionally, since the proximity of the TH location may have an impact on the physical and mental well-being of the participants, the distant location of the TH facility, which required an off-unit trip for participants in this study also may have compromised the outcome. Further, given the wide range of TH environments available, including hospital gardens, agricultural stations, and farms [45-46], limiting the TH only to one facility may have limited various social interactions that may have potential effects on the outcome.

## Conclusions

This study supports a promising avenue to further explore the potential benefits of adding TH in the treatment of MDD and TR-MDD. Previous small group studies have shown improvement in the overall clinical symptoms of depression by TH. This study extends this positive trend in the treatment of MDD as a potential adjuvant to ECT and antidepressants. We also assert that the recently increased participation in gardening among the general population points to a non-stigmatizing and potentially socially reinforcing context, increasing the promise of TH benefits among treatment enhancing options for those affected by MDD and TR-MDD. Specifically noteworthy is the improvement on the Role Limitations-Physical Health (RLPH) subscale in the intervention group who received TH along with ECT/antidepressants. Although further large cohort studies with well-defined parameters are needed to explore specific mental, emotional, physical, and social benefits of TH on MDD, this study provides the basic premise for consideration of various TH settings, including convenient inpatient gardens to achieve therapeutic benefits through plants and nature within patient settings.
